# Incidence of childhood psychiatric disorders in India

**DOI:** 10.4103/0019-5545.49449

**Published:** 2009

**Authors:** Savita Malhotra, Adarsh Kohli, Mehak Kapoor, Basant Pradhan

**Affiliations:** Department of Psychiatry, Postgraduate Institute of Medical Education and Research, Chandigarh - 160 012, India

**Keywords:** Incidence, childhood psychiatric disorders, epidemiology

## Abstract

**Background:**

Studies on incidence of childhood mental disorders are extremely rare globally and there are none from India. Incidence studies though more difficult and time consuming, provide invaluable information on the pattern and causes of occurrence of mental disorders allowing opportunity for early intervention and primary prevention.

**Aim:**

This study aimed at estimating the incidence of psychiatric disorders in school children.

**Materials and Methods:**

A representative sample of school children was assessed through a two stage evaluation process involving teacher's rating (N=963) and parent rating (N=873). Children who scored below the cut-off for psychiatric disorder (N=727) on both the screening instruments were re-contacted six years later. 186 children and their families were personally available for reevaluation. All the children and their parents were re-assessed on Parent Interview Schedule; Strengths and Difficulties Questionnaire: and detailed clinical assessment by a psychiatrist. Psychiatric diagnosis was made as per ICD 10 criteria. Data on children who were found to have psychiatric disorder were compared with those who did not have psychiatric disorders.

**Results:**

20 children out of 186 followed up had psychiatric disorder giving the annual incidence rate of 18/1000/yr. Children who had disorder at follow-up did not differ from those who did not on age, gender and psychological (temperament, parental handling, life stress and IQ) parameters at baseline.

**Discussion:**

Incidence figures cannot be compared due to lack of any comparable studies. Factors associated with occurrence of new cases of psychiatric disorder and implications for future studies are discussed.

## INTRODUCTION

Prevalence of mental disorders among children has been reported to be 14-20% in various studies.[1] According to World Health Report (2000), 20% of children and adolescents suffer from a disabling mental illness worldwide[2] and suicide is the third leading cause of death among adolescents.[3] The issue of childhood psychiatric morbidity is more serious in middle and low income countries because these countries have a much larger proportion of child and adolescent population; much lower levels of health indices; poorer infrastructure and resources to deal with problems.

In recent years, there have been several population studies giving fairly reasonable estimates on the prevalence of child and adolescent mental disorders (CAMD) in low and middle income countries. Reported rates are 17.7% in 1-15 yrs old in Ethopia;[4] 15% among 5-10 yrs olds of Bangladesh;[5] 12.7% in 7-14 yrs olds urban Brazilian school sample[6] and 7% in 7-14 yrs rural Brazilian school[7] and 6.9% in 4-17 yrs. Puerto Rican community based sample.[8] Studies from India have revealed the prevalence rates to be 12.5% in 0-16 yrs community based sample from Bangalore;[9] 9.4% in 8-12 yrs olds from a community sample in Kerala[10] and 6.3% in 4-11 yrs old school children in Chandigarh.[11] Overall rates of CAMD in India and other middle and low income countries range between 6%-15% which are on the lower side as compared to reported rates from certain western countries such as Canada 18.1%,[12] Germany 20.7%,[13] Switzerland 22.5%,[14] USA 21%.[15] It is also known that many more children have problems that can be considered “sub threshold” since these may not meet the diagnostic criteria.

There are very few studies on incidence of CAMD worldwide. Occasional studies available looked at incidence of single, specific disorder such as depression, OCD, anxiety, PDD etc. Incidence of major depressive disorder and dysthymia was reported as 3.3% and 3.4% respectively in a study from the US;[16] one year incidence of OCD was 0.7% and sub clinical OCD was 8.4%.[17] One year incidence of phobic disorder was 0.4%, whereas sub syndromal and sub threshold phobia was 8% and 16.9% respectively.[18]

Although incidence studies are more difficult, more expensive, less feasible and more time consuming, but these are more valuable as these provide the possibility of studying the risk factors and understanding the context of development of disorders; and knowledge of early manifestations of illness, indicating intervention opportunities for primary prevention.

Most CAMD have typical ages for development and presentation, these tend to continue into adulthood, and further several adult mental disorders have their antecedents in childhood.[19] It is therefore, extremely important that the incidence and context of occurrence of CAMD are studied to identify and to undertake preventive intervention early in the course of illness.

This study aimed at estimating the incidence of psychiatric disorders in school children in the city of Chandigarh, India.

## MATERIALS AND METHODS

**Figure 1:**
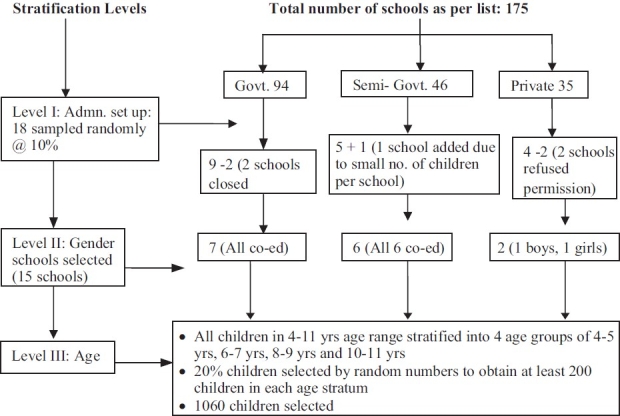
Sampling procedure involved a three stage stratified random sampling of all the registered schools in Chandigarh and then of all the children in specified age range in the selected schools

**Figure 2:**
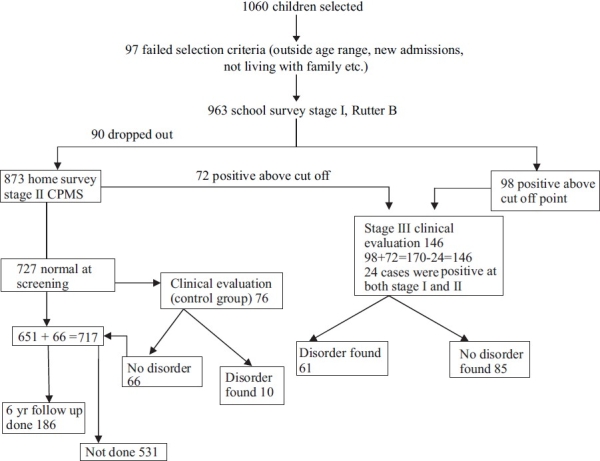
Study procedure

Our earlier study of prevalence of psychiatric disorders in school children in Chandigarh[11] formed the baseline for the present incidence study. The prevalence study involved a multistage, multi informant assessment of a stratified random sample of 4-11 yrs olds school children, and gave a prevalence figure of 6.33%. Detailed procedure of sampling and methodology of prevalence study are already published[11] [Figures 1 and 2].

873 children, who were evaluated by teacher's rating using Rutter B scale and parent's rating using Childhood Psychopathology Measurement Schedule (an Indian adaptation of CBCL) respectively, identified 146 children who scored above the cut off points for possible psychiatric disorder; and remaining 727 children scored below the cut-off points on both the screening questionnaires i.e. teacher's rating and parent's ratings. These children were considered as “normal group” and constituted the sample for the follow up study. At the baseline, all children and their parents preferably mother were interviewed to assess children's temperament using Temperament Measurement Schedule[20] and life events using Life Events Scale for Indian Children[21] and parental handling using Parental Handling Questionnaire.[22] Temperament Measurement Schedule is an Indian adaptation of Thomas and Chess's[23] schedule for temperament assessment and assessed nine dimensions of temperament i.e. activity, intensity, approach-withdrawal, adaptability, mood, persistence, rhythmicity, threshold of responsiveness and distractibility. This is a bilingual semi-structured scale with five items for each of the nine dimension and provides averaged scores for each temperament dimensions. These nine temperaments are further reduced to five factors on factor analysis. Scores for each of the five factors are obtained by summation of scores on the constituent dimensions. And these are: Sociability (includes approach-withdrawal, adaptability and threshold of responsiveness); Emotionality (includes mood and persistence); Energy (includes activity and intensity); Distractibility and Rhythmicity.

Parental Handling Questionnaire:[22] This scale is an Indian adaptation of Parent Bonding Instrument.[24] It measures parental handling on two parenting dimensions: parental care (10 items) and parental control (4 items), on a three point scale 0 (no), 1 (sometimes) and 2 (yes). Higher score indicates higher care and higher control and vice versa. The scale has adequate reliability and validity.

Life Events Scale for Indian Children:[21] This is an Indian adaptation of British Life Events Inventory,[25] comprising of 50 items. Life Events Scale for Indian Children[21] provides stress scores for each item given and validated for Indian socio-cultural context. The scale has high test-retest reliability and inter-rater reliability (+ 0.89 and + 0.92) respectively. In addition the scale provides a measure of number of life events in the last one year or during the life time. Subjective evaluation of each of these events i.e. stressfulness score (0, 1, and 2) is assessed and summated to give subjective stress score. Scale has satisfactory reliability and validity.

All subjects in the normal group (N=727) were re-contacted after 6 yrs of initial evaluation. They were in the age range of 10-17yrs. Multiple follow-up attempts were made to contact every family through telephone calls, letters and home visits over a period of three months. Minimum number of attempts was five per subject. Efforts were made to follow the trail of their changed residence or changed school and were given up only when it was not possible to physically contact the subject within the catchment area of the city of Chandigarh. Reassessment was done on Parent Interview Schedule PIS[26] and Strengths and Difficulty Questionnaire SDQ.[27] Informant in all cases was a parent preferably mother. Children were also directly assessed. Evaluation questionnaires were administered by a clinical psychologist or a psychiatrist. Children scoring above a cut off score on SDQ (≥ 14) were clinically examined by a psychiatrist at home or at clinic.

Parent Interview Schedule (PIS)[26] provides assessment of parent's attitude and ways of dealing with the problems in the child. PIS consists of 19 questions, out of which 9 are related to the child's behaviour in general, and other 10 questions covered 10 problem behaviours i.e. emotional problems, behavioural problems, disruptive behaviour, bed wetting, conduct problems, somatic complaints, school refusal, stealing etc. We used the Indian adaptation of the upgraded version of the Parent Interview Schedule (PIS).

Strengths and Difficulties Questionnaire (SDQ)[27] is a brief behavioural screening questionnaire that provides balanced coverage of children and young people's behaviour, emotions and relationships. The questionnaire explores into 25 attributes, some positive and others negative and these 25 items are divided into 5 subscales of 5 item viz. hyperactive scale, emotional scale, conduct problems, peer problems, and social problems. Hindi version of SDQ is available and has been used. Scores 0-13 are considered normal, 14-16 are borderline and 17-40 are abnormal. SDQ is a widely used scale in epidemiological studies on children and adolescents world over.

Baseline study data on CPMS and Rutter B scores, Temperament, Parental Handling Questionnaire, Life Events Scale for Indian Children was analyzed and used for comparative analysis.

186 subjects (25.6%) could be personally re-contacted and were re-evaluated. All cases underwent a clinical psychiatric evaluation by a qualified psychiatrist. Detailed clinical history from parents and psychiatric examination of the child was done. Psychiatric diagnosis was made as per ICD-10 criteria.

Figures show the complete process of sampling and evaluation.

Incidence rates were calculated on the basis of number of children in whom psychiatric disorder was reported any time during the six yrs period. Baseline data on age, gender, socioeconomic status (SES), temperament, parental handling and life events were analyzed separately for children with disorder and those without disorder and was compared.

## RESULTS

20 children out of 186 followed-up were identified to have psychiatric disorder. This gives the incidence rate of 18/1000/yr (0-37, 95% CI). There were 9 males, 11 females. Baseline and current mean age of the disorder group was 7.47 yrs (±2.29) and 13.2yrs (±2.38), whereas of those with no disorder was 7.20 yrs (±2.37), and 13.38 yrs (±2.31) respectively.

Table 1 shows the comparison of the normal group of children (N=186) who were followed up after 6 yrs, and those (N=561) who could not be followed up, on age, gender and socio-economic status to see if there was any sampling bias. Drop out group did not differ from the follow up group on age and gender whereas significantly larger number of children from high SES could not be followed up. Thus, the follow up group had significantly more children from middle and lower SES.

**Table 1:** Sociodemographic comparison of follow-up group with those who were not followed up

	Follow up group N=186	No follow up group N= 531	t/χ^2^
Gender			
Males (%)	86 (46.24)	43 (45.76)	χ^2^ 0.01
Females (%)	100 (53.76)	288 (54.23)	
SES*			
Lower (%)	52 (27.9)	110 (20.72)	χ2 18.4**
Middle (%)	53 (28.5)	95 (17.89)	
Higher (%)	81(43.6)	326 (61.39)	
Mean age (SD)	7.49 yrs (2.29)	7.41 yrs (2.10)	t 0.42

** *p*<.01

Table 2 gives the description of psychiatric diagnoses in the normal group. Diagnosis in 12 subjects fell into adult disorders category and in 8 it was disorder with onset specific to childhood. Children with psychiatric disorder were compared with those who did not have psychiatric disorder on socio-demographic [Table 3] and psychological variables [Table 4] at baseline. Children who developed disorder did not differ from those who did not on age, gender, socioeconomic factors as well as psychological variables of temperament, parental handling, life events and IQ.

**Table 2:** Psychiatric diagnosis in the normal sample

ICD-10 code	Diagnosis	N=20
F 32	Depressive episodes	3
F 41.2	Mixed anxiety and depressive disorder	3
F 42.2	OCD, mixed thoughts and acts	1
F 43.2	Adjustment dis. brief depressive reaction	3
F 60.4	Histrionic personality dis.	1
F 63.2	Pathological stealing	1
F 81.3	Specific developmental disorder of scholastic skills	2
F 90	ADHD	2
F 91	Conduct disorder	1
F 93.0	Emotional disorder with onset specific to childhood	2
F 94	Childhood disorder of social functioning	1

**Table 3:** Sample description of follow up group N=186

	No disorder (N=166)	Disorder (N=20)	t/χ^2^*
Mean age (SD)	7.47 (2.29)	7.20 (2.37)	t=0.48
Sex			
Male (%)	77 (46.39)	9 (45)	χ^2^ .01
Female (%)	89 (53.61)	11(55)	
SES			
Low (%)	52 (31.33)	3 (15)	χ^2^ 2.3
Middle (%)	45 (27.11)	7 (35)	
High (%)	69 (41.57)	10 (50)	

* not significant

**Table 4:** Comparison of baseline parameters of groups of children who developed psychiatric disorders at follow up with those who did not

	N=166 (No disorder)	N=20 (Disorder)
Screening scores		
Rutter B*	2.36 (2.24)	4.10 (2.69)
CPMS	3.33 (2.80)	4.70 (2.64)
Temperament scores	9.38 (.88)	9.40 (1.12)
Sociability	6.38 (.49)	6.32 (.42)
Emotionality	6.31 (.59)	6.60 (.51)
Energy rythmicity	2.85 (.28)	2.81 (.26)
Distractability	3.19 (.38)	3.26 (.38)
Parental handing scores		
Parental care	13.57 (3.73)	12.70 (2.97)
Parental control	4.85 (3.66)	4.60 (1.43)
Life events and stress scores		
No. of life events	5.50 (1.93)	6.20 (2.09)
Subjective stressfulness	6.77 (3.52)	8.40 (3.89)
Quantitative score	209. 76 (83.61)	235.65 (85.4 2)
I. Q.	97.93 (15.93)	97.38 (7.52)

* t 3.2; p<.05

## DISCUSSION

Globally research literature on the incidence of childhood psychiatric disorders is scarce. Incidence studies, by definition, are longitudinal and prospective. Importance of incidence studies in general, and in child and adolescent psychiatry in particular, cannot be over-emphasized. Many child psychiatric disorders may not present clear-cut disease like entities and change over time. There has been great difficulty in determining the point at which behaviour or a combination of behaviors will be considered as pathological which may again be dependent on the age or the socio-cultural context of the child. What is pathological at one age may not be so at another and what is pathological in one culture may not be so in another. Only longitudinal, prospective studies can resolve these issues and such studies further provide the opportunity to examine the pattern of distribution of childhood psychiatric disorders in the community setting which in turn will guide our future endeavors for early intervention.

There is no study on incidence of childhood psychiatric disorders in India. The present study is the first prospective, longitudinal, 6 years follow-up study in order to estimate the incidence and the pattern of distribution of childhood psychiatric disorder in school children.

In our study, the incidence of psychiatric disorders in the normal children group came out to be 18 per thousand per year (95% confidence interval 0-37) which means that we can say by 95% confidence that the incidence rate is not higher than 37/1000/yr. Since the figure 18 lies almost at the midpoint, we may say that this incidence rate perhaps lies at the lower limit of actual incidence. One of the reasons for a wide confidence interval could be a small sample size. In fact, it was a very difficult task to re contact children and their families after a gap of 6 yrs.

Incidence studies are extremely rare even in the other parts of the world. In a collective expert report, published in 2007, on mental illness in children and adolescents in 2001-2002, incidence of mental illness in children in France was reported to be 1 in 8.[28] In this report, the incidence of anxiety disorders was 5%, hyperactivity 1-2%, mood disorder 3% (13-19 yrs olds), and autism and schizophrenia 1%. In another study, incidence of somatoform disorders was reported to be 12% (lifetime incidence) and 7% (12 month incidence) among adolescents in a general population sample in Germany.[29] There are reports that ethnicity is one of the factors in prevalence of psychiatric disorders in children. In a report of a survey of mental health of children and adolescents in Great Britain,[30] it was shown that the overall rate of mental disorder, among 5-15 yrs olds, as per ICD-10, was 10% (including those who had more than one disorder). Differential rates as per ethnicity were: 10% among white children, 12% of black children, 8% among Pakistanis and Bangladeshis and 4% among the Indian children. Considering this and other findings of relatively lower prevalence rates in Indian children, it is likely that the incidence rate will also be lower and the incidence of 18/1000/yr in our study could actually be a true reflection.

Since no comparative data on incidence of childhood psychiatric disorders are available from India, we could not compare our findings.

In this study, the dropout rate has been considerable despite extensive and best possible methods employed to trace subjects. The reasons for this drop out could be that Chandigarh city has high proportion of migrant population and in our study majority of children were from families of government employees and slums. A large proportion of government employees come to Chandigarh on a time bound deputation from three adjacent states, i.e. Haryana and Punjab (of which Chandigarh is the joint capital) and Himachal Pradesh, after which they relocate to their parent state. Also the Chandigarh Administration has a policy to relocate slums making it impossible to re contact families who lived in slums at the time first study.

In the present study, sample consisted of school children. Thus the methodology of our study would have excluded children with severe psychopathology and dysfunctions, such as autism, severe learning disorders, psychotic or severe disruptive behaviour disorders etc. Also transient psychiatric disturbances such as brief episodic adjustment disorders could have been missed during the six years of follow up duration due to possible recall problems.

Looking at the pattern of psychiatric disorders in this sample, it is apparent that 10 (50%) children fell into the category of neurotic, stress related and affective disorders; and 2 (10%) children had personality and behaviour disorders. These conditions are basically adult disorders which had onset during childhood. Only 8 (40%) children presented with disorders that have onset specific to childhood. It has been reported that the peak hazard rate for major depression, mania, OCD, phobias and drug and alcohol disorders was in childhood or adolescence.[32] Kim-Cohen *et al*, in their follow back study from New Zealand reported that 50% of adult psychiatric disorder cases had onset by age of 15.[33] These findings point to the fact that there is considerable morbidity in childhood due to onset of adult psychiatric disorders. Costello *et al*,[31] in their updated review of epidemiology of childhood psychiatric disorders have opined “that onset before adulthood may be a characteristic of the majority of adult psychiatric disorders”. Early intervention for these has the potential to substantialy alter the developmental course of these adult disorders, significantly reducing the morbidity.

Effort was made to examine the social psychological correlates of psychiatric disorders and to find out if the group who developed psychiatric disorder differed from those who did not at the baseline evaluation. Children with disorder (N=20) and with no disorder (N=166) were compared on sociodemographic and psychological variables at baseline [Table 3]. Children with disorder and with no disorder were comparable on age, gender and SES.

The two groups differed at screening on, Rutter B scores which was higher in disorder group although it was much below the cut-off levels. There was no difference on child's temperament, parental handling, life stress and IQ levels between the two groups. It was found that there was no age or gender difference.

Relationship between age, gender and psychiatric disorders of childhood and adolescence is different for different groups of disorders. PDD's, SLD's, ADHD, aggression, enuresis have early onset and are more common in males; whereas depression, anxiety, neurotic and stress related disorders have later age of onset and are more common in females. It has been reported that most of the childhood onset disorders are more common among males, whereas, most of adolescent onset disorders are more in females.[34] In this study since the mean age of the sample was about approx. 13 yrs in both the groups, no gender difference was found for general psychopathology.

Disorder group of children had higher scores on Rutter B although it was well below the cut-off level for possible psychiatric disorder. It may indicate that higher scores at screening or in other words problems in school adjustment should alert one to the possibility of psychiatric disorders in the long term.

Several risk factors described for mental disorders in children have been summarized in a review[35] and these have been categorized as individual determinants such as gender, temperament and neurobiological risks, disabilities etc; family determinants including family's structure, its functioning, parenting styles, attachment patterns, parental mental health, physical abuse and punishment etc; and social and community determinants such as socioeconomic deprivation, ecological factors, conflict and war etc.

Although temperament has been considered to be one of the important vulnerability factors for psychiatric disorder in children, there was no relationship found in this study. Similarly, parental handling assessed as amount of care given or control exercised on the child was not different between the two groups. One of the reasons for the negative findings could have been a small number of subjects in the disorder group. In this study number of life events, overall stress score and subjective stressfulness scores were higher in the disorder group than those in the no disorder group but the differences were not significant statistically. However, the trend is significant. It has been reported that high levels of life time exposure to adversity is casually implicated in the onset of depressive and anxiety disorders in children.[36] It is likely that the trends exhibited in the expected direction did not reach significance due to small numbers in the sample.

### Strengths of our study

It is the first systematic study done in India on incidence in child psychiatry, with a careful and sound research design, on a representative sample of school children chosen through stratified random sampling procedure, overcoming several methodological limitations that are generally recognized. It is a prospective longitudinal study and assessments were done thoroughly by qualified psychiatrists and by well-structured and standardized scales, using standard (ICD 10) diagnostic criteria. Assessment scales included life-time versions of symptoms. Follow up strategy had been intensive door to door survey.

### Limitations

The sample size in our study is relatively small. Also the dropout rate is high for the reasons already described. As a part of the design of this study, only school population was inducted and thus we might have missed children with severe psychopathology and dysfunctions, e.g. pervasive development disorders, severe mental retardation etc. or those who do not enter the school system, or drop out early. Also transient psychiatric disturbances in form of brief episodic mental disorders could have been missed in our study considering that the assessments were done only at two points of time, six years apart, in a prospective longitudinal design. Other limitation might be the retrospective recall bias inherent to our methodology.

## CONCLUSION

Our study examined the incidence of childhood psychiatric disorders in the community setting and the rate was 18/1000/yr; the rate could be said to range between 18-37/1000/yr. The study highlights the need for such large scale studies to understand the rates and pattern of causation. Difficulties encountered in studies of large scale, prospective, longitudinal samples were amply encountered in our study as well.
